# Characterization of Lysophospholipase D Activity in Mammalian Cell Membranes

**DOI:** 10.3390/cells13060520

**Published:** 2024-03-16

**Authors:** Yuhuan Xie, Krishna M. Ella, Terra C. Gibbs, Marianne E. Yohannan, Stewart M. Knoepp, Pravita Balijepalli, G. Patrick Meier, Kathryn E. Meier

**Affiliations:** 1 Department of Cell and Molecular Pharmacology and Experimental Therapeutics, Medical University of South Carolina, Charleston, SC 29425, USA; 2 Department of Pharmaceutical Sciences, College of Pharmacy and Pharmaceutical Sciences, Washington State University, Spokane, WA 99202, USA; pravita.balijepalli@wsu.edu

**Keywords:** lipid mediators, phospholipases, signal transduction

## Abstract

Lysophosphatidic acid (LPA) is a lipid mediator that binds to G-protein-coupled receptors, eliciting a wide variety of responses in mammalian cells. Lyso-phospholipids generated via phospholipase A_2_ (PLA_2_) can be converted to LPA by a lysophospholipase D (lyso-PLD). Secreted lyso-PLDs have been studied in more detail than membrane-localized lyso-PLDs. This study utilized in vitro enzyme assays with fluorescent substrates to examine LPA generation in membranes from multiple mammalian cell lines (PC12, rat pheochromocytoma; A7r5, rat vascular smooth muscle; Rat-1, rat fibroblast; PC-3, human prostate carcinoma; and SKOV-3 and OVCAR-3, human ovarian carcinoma). The results show that membranes contain a lyso-PLD activity that generates LPA from a fluorescent alkyl-lyso-phosphatidylcholine, as well as from naturally occurring acyl-linked lysophospholipids. Membrane lyso-PLD and PLD activities were distinguished by multiple criteria, including lack of effect of PLD2 over-expression on lyso-PLD activity and differential sensitivities to vanadate (PLD inhibitor) and iodate (lyso-PLD inhibitor). Based on several lines of evidence, including siRNA knockdown, membrane lyso-PLD is distinct from autotaxin, a secreted lyso-PLD. PC-3 cells express GDE4 and GDE7, recently described lyso-PLDs that localize to membranes. These findings demonstrate that membrane-associated lyso-D activity, expressed by multiple mammalian cell lines, can contribute to LPA production.

## 1. Introduction

Lysophosphatidic acid (LPA) refers to a class of small lipid mediators that bind to G protein-coupled receptors (GPCRs) [1,2]. These receptors, LPARs 1–6, are widely though differentially expressed in mammalian cells. LPA can induce numerous responses in target cells, including activation of phospholipases C (PLC) and D (PLD), calcium mobilization, activation of mitogen-activated protein kinases, tyrosine phosphorylation, cell spreading, cell migration, cell attachment, and proliferation [3,4]. It was first shown that LPA could be released by activated platelets [5]. It is now clear that many cell types can produce LPA [2,6,7,8], supporting its role as both an autocrine and paracrine mediator. However, there are multiple enzymatic pathways potentially involved in LPA generation, and their respective roles have not been fully delineated.

One potential route for LPA production involves the sequential actions of PLD and PLA_2_ on phosphatidylcholine (PC). PLD is activated in mammalian cells in response to many stimuli, including growth factors and agonists binding to G-protein-coupled receptors [9]. The product of the PLD reaction, phosphatidic acid (PA), may itself serve as a second messenger molecule. This has been most clearly demonstrated in plants [10]. Another role of PA is as a precursor for other messengers. First, lipid phosphate phosphatases (LPPs) can metabolize PA to diglycerides (DG), potentially providing diacylglycerol to activate protein kinase C isoforms [11]. Second, metabolism of PA by PLA_2_ can potentially yield LPA. PA-utilizing PLA_2_s have been described [12], and calcium-independent iPLA_2_ can use PA as substrate [13]. Evidence for the PLD/PLA_2_ route has been presented [14,15], indicating that this pathway can result in LPA production.

A second potential route for LPA generation is via the sequential actions of PLA_2_ and a lyso-PLD [2,16]. Mammalian lyso-PLD activities were first characterized in the 1970s [17,18,19,20,21]. A lyso-PLD present in microsomes from various mammalian tissues was reported to preferentially utilize 1-alkyl-linked lyso-PCs [19,20]. Lyso-PLD activity toward alkyl-linked substrates was also reported in intestinal epithelial cells [22] and in *Leishmania donovani* [23]. In the 1980s, lyso-PLDs were particularly considered with respect to metabolism of platelet activating factor (PAF), alkyl-linked PCs with short chain esters at the *sn*-2 position [24,25,26,27,28,29]. Subsequently, the roles of secreted lyso-PLDs in LPA generation were addressed. A lyso-PLD present in mammalian plasma, serum, and other biological fluids was shown to prefer lyso-PCs with polyunsaturated acyl substituents [30,31,32,33,34]. A lyso-PLD secreted by rat adipocytes utilized acyl- and alkyl-linked lyso-PCs [35]. The lyso-PLD present in serum was eventually identified as autotaxin (ATX), a motility-stimulating factor with phosphodiesterase activity [2,36,37]. More recently, two additional lyso-PLDs were identified: GDE4/Gdpd1 [38,39] and GDE7/Gdpd3 [38,40]. GDE4 and GDE7 are found in the membrane fraction when over-expressed in mammalian cells [39,41]; GDE4 can localize to the perinuclear/Golgi region [38]. GDE4 and GDE7 can hydrolyze both acyl- and alkyl-linked substrates but prefer acyl-linked species [38,41].

There are at least two additional pathways for LPA generation. One is phosphorylation of monoglyceride. Analogously, phosphorylation of sphingosine by sphingosine kinase generates sphingosine-1-phosphate [42], a lipid mediator that binds to GPCRs that are closely related to LPA receptors. Another potential pathway is via the reverse reaction of LPA acyltransferase (LPAAT), generating acyl-CoA and LPA from PA [43].

From a cell signaling perspective, it is important to understand the origin of LPA. Previous work from our lab established that mitogenic agonists can increase LPA levels both within cells and in the extracellular space [8]. LPA that is either generated extracellularly by autotaxin or added exogenously to cells is capable of activating the GPCRs that mediate LPA actions (LPARs 1–6). Autotaxin can facilitate presentation of LPA to some of its receptors [44]. The access of LPA to LPARs has been modeled for some LPARs [45,46]. In these models, there is controversy concerning whether LPA accesses its receptors via the membrane or the extracellular aqueous phase. It is conceivable that LPA generated by membrane-localized lyso-PLDs may access receptors in a facilitated manner or interact with receptors internalized into an intracellular compartment. Thus, the location in which LPA is produced may impact signal transduction mediated by LPA receptors.

In the current study, we demonstrate that a lyso-PLD activity that utilizes acyl- and alkyl-PCs is widely expressed in various mammalian cell lines. This enzyme activity is localized to cell membranes.

## 2. Materials and Methods

### 2.1. Cell Culture

PC12K (rat pheochromocytoma) [47,48] and A7r5 (rat vascular smooth muscle) [49] cells were maintained in Dulbecco’s modified Eagle’s (DME) medium supplemented with 10% fetal calf serum. Rat-1 HIR (rat fibroblast) cells expressing the human insulin receptor were maintained in DME/F12 (1:1) medium supplemented with 10% fetal calf serum (UBI) and 0.25 µM methotrexate [50]. PC-3 (human prostate cancer) cells were maintained in F12K medium [51] (for enzyme assays) or RPMI 1640 [52] (for proteomics experiment) supplemented with 10% fetal calf serum. SKOV-3 and OVCAR-3 human ovarian cancer cells, obtained from the American Type Culture Collection, were maintained in McCoy’s 5a medium with 10% fetal calf serum and RPMI 1640 medium with 1 mM sodium pyruvate, 0.01 mg/mL bovine insulin, and 20% fetal calf serum, respectively. 

### 2.2. Preparation of Cell Membranes

Membranes were prepared as described previously [53]. Briefly, cells were washed with PBS, resuspended in ice-cold lysis buffer (20 mM HEPES [pH 7.5], 80 mM ß-glycerophosphate, 10 mM EGTA, 2 mM EDTA, and 5 mM dithiothreitol), sonicated, and sedimented at 100,000× *g* for 20 min at 4 °C. The pellet (membrane fraction) was resuspended in lysis buffer. In some experiments, the supernatant (cytosol fraction) was used. Protein concentrations were determined using a Bradford assay.

### 2.3. Phospholipase Assays

In vitro assays, originally optimized for PLD activity, were carried out as described previously using BODIPY-PC (B-PC; 2-decanoyl-1-(O-(11-(4,4-fluoro-5,7-dimethyl-4-bora-3a,4a-diaza-s-indacene-3-propionyl)amino)undecyl)-sn-glycero-3-phospho-choline, Molecular Probes, Eugene, OR, USA catalog #D-3771), a 1-alkyl 2-acyl phosphocholine, as substrate [54]. The final reaction mix (12.5 µL) contained 0.1 mM B-PC, 150 mM NaCl, 200 µM of octylglucoside, 25 mM HEPES (pH 7.0), 5 mM EGTA, 1 mM EDTA, 40 mM of ß- glycerophosphate, 1 mM dithiothreitol, and 1% (*v*/*v*; 0.135 M) n-butanol. The reaction, initiated by the addition of membranes (10 µg of protein), was carried out for 60 min at 30 °C. Reaction products were separated using thin-layer chromatography (TLC) developed with chloroform/methanol/water/acetic acid (45:45:10:2) [54]. Plant (cabbage) PLD, *Streptomyces* PLD, and bee venom PLA_2_ were from Sigma (St. Louis, MO, USA). For some experiments, alternative fluorescent substrates were used: BODIPY-lyso-PC (B-lyso-PC; Molecular Probes, catalog #D-3772) and BODIPY-C_12_-fatty acid (Molecular Probes, catalog #D-3823). In some cases, BODIPY-labelled phospholipase substrates (e.g., phosphatidic acid, phosphatidylethanol, and lyso-PC) were generated from B-PC using partially purified enzymes or cell membranes separated using TLC, as described above, extracted from silica gel using ethanol/acetic acid (100:1), dried, and resuspended for use in phospholipase assays carried out in the absence of butanol. Non-fluorescent lyso-phospholipids, used in competition assays, were obtained from Cayman Chemical Company (Ann Arbor, MI, USA) and Avanti Polar Lipids (Alabaster, AL, USA). Fluorescent products were separated using TLC and then imaged using a FluorImager (Molecular Dynamics, Sunnyvale, CA, USA). Data were quantified using either FluorImager (V1) or NIH Image (J1) software. For some experiments, membranes were incubated with [^3^H]-lyso-PAF (NEN Life Sciences, Boston, MA, USA; 1 µCi/mL) rather than a fluorescent substrate. Products were separated on oxalate-impregnated TLC plates [8] and visualized using autoradiography. Graphical presentations were done using GraphPad Prism (La Jolla, CA, USA, V. 6.0)

### 2.4. HPLC Separation of Lipids

PC12K membranes were incubated with B-PC or B-lyso-PC, as described above. As a positive control, B-PC was incubated with plant PLD followed by bee venom PLA_2_. The reaction mixtures in their standard reaction volumes, as described above, were applied to a SEP-PAK silica cartridge (Waters Assoc., Inc. Milford, MA, USA), from which phospholipids were eluted with 10 mL chloroform/methanol (1:1). The eluate was dried under nitrogen and dissolved in 20 µL sample solvent (chloroform/methanol/H_2_O/acetic acid; 35:55:10:3). Lipid separation was carried out using a Gilson (Middleton, WI, USA) HPLC 715 system. The sample solvent was used as the mobile phase. An Si 60 (4.6 × 260 mm) column (Lichrosorb) was used at a flow rate of 0.6 mL/min. The UV detector wavelength was 254 nm.

### 2.5. Over-Expression of PLD2

A hemagglutinin-tagged human PLD2 construct was generated in our group from PC-3 cell RNA using RT-PCR and verified using sequencing [55]. The construct was ligated into PBluescript vector (modified for mammalian expression) under the control of the CMV promoter. PC12K cells were transiently transfected using Lipofectamine 2000 (Gibco/BRL). Transfection efficiencies of ~50% were achieved, as quantified using Fluorescence-activated cell sorting (FACS) analysis of cells transfected with vector encoding green fluorescent protein (GFP).

### 2.6. Radioenzymatic Assay for LPA

An assay for bulk LPA was carried out according to a published method [56]. Briefly, this assay converts unlabeled LPA to [^14^C]-PA using bacterially expressed mammalian LPA acyltransferase (LPAAT). The LPAAT construct [57] was kindly provided by Dr. J.S. Saulnier-Blache (INSERM, Toulouse) with the permission of Dr. K. Kume (University of Tokyo). Membranes were incubated as described for the fluorescent phospholipase assays, but using 0.2 µM unlabeled C_18_-lyso-PAF (Avanti) as substrate rather than a fluorescent lipid. The reaction was carried out for 40 min at 30 °C. LPA was then extracted from the reaction mixture using chloroform/methanol/HCl, as described previously [8,54]; the organic phase was then washed once with 0.5 M NaCl. Lipid extracts were dried and then suspended in assay buffer containing Tween 20, [^14^C]-oleoyl-CoA (NEN Life Sciences; 20 µCi/mL), and microsomal membranes from *E. coli* expressing murine LPAAT [56]. As a positive control, 18:1 LPA (20 pmole), LPAAT, and [^14^C]-oleoyl CoA were added to lipid extracted from a mock reaction lacking mammalian membranes and then incubated as described above. Following incubation, lipids were extracted and separated using TLC. [^14^C]-PA was localized and imaged using autoradiography.

### 2.7. Autotaxin Knockdown

To assess autotaxin mRNA levels, total RNA was prepared using an RNeasy Mini Kit (Qiagen, Redwood City, CA, USA) and treated with deoxyribonuclease I. The primers for human autotaxin were forward 5′-CACTTGCTCAGCCTGCCGACAAG[FAM] G-3′; reverse 5′-TGCAGCTCTCCTCGTTGTCA-3′; and human β-actin certified LUX^TM^ primer set (Invitrogen, Carlsbad, CA, USA). RT-PCR amplification was carried out in a volume of 25 µL according to the manufacturer’s instructions for Superscript TM III Platinum^®^ One Step Quantitative RT-PCR System (Invitrogen, Carlsbad, CA, USA). Data were acquired using Gene Amp^®^ 5700 (PE Biosystems, Foster City, CA, USA). Fold-change in expression was calculated using the 2^ΔΔCT^ formula, where ΔΔCT stands for the difference in ΔCTs between the compared samples and the ΔCT of any single sample equals CT of autotaxin for that sample minus CT of the reference gene, β-actin, from the same sample. PCR products were verified after separation on an agarose gel with ethidium bromide.

Silencing RNA (siRNA) against human autotaxin was custom-designed and synthesized (Ambion, Inc. Austin, TX, USA). The sequence specificity was confirmed (using a BLAST search) to be unique from other known human genes. The target sequence started at position 171 of human autotaxin (AAGGTCCTCCTACAGTGCTAT). The siRNA duplex was delivered to the cells using Lipofectamine 2000 reagent (Invitrogen, Carlsbad, CA, USA) according to the manufacturer’s instructions. Briefly, OVCAR-3 cells were plated at a density of 70,000 cells/well in 6-well plates and grown for 24 h. For each well, 5 µL of Lipofectamine 2000 was incubated with 250 µL of OPTI-MEM^®^ medium for 5 min. Subsequently, a mixture of 1 µL (100 pmol) of the annealed siRNA in 250 µL OPTI-MEM^®^ medium was added. The final mixture was incubated for 20 min at room temperature and then added to each well, resulting in a siRNA concentration of 33.3 pmol/mL. Cells were used for experiments 72 h after transfection.

### 2.8. Proteomics Analysis

Proteomic data for GDE4 and GDE7 were extracted from an archived dataset generated in a global proteomics analysis [52]. Briefly, PC-3 cells (pre-incubated with a scrambled siRNA) were grown for 48 h, serum starved for 24 h, and then incubated in triplicate in fresh serum-free medium for 3 h. Cell lysates were collected, subjected to trypsin proteolysis, desalted, and processed for liquid chromatography/mass spectrometry. Proteomics data were acquired and analyzed as described [52] and further processed using Microsoft Excel (Microsoft 365 MSO V.2401) and Graph Pad PRISM (Prism 10 for Windows 64-bit). Proteins found in at least 2 out of 3 replicates were considered detectable.

## 3. Results

### 3.1. Metabolism of BODIPY-PC in Membrane Preparations

Previous work from this laboratory utilizing a fluorescent BODIPY-alkyl linked glycerophosphocholine (B-PC) as a substrate for PLD [53,58] showed that a product tentatively identified as BODIPY-LPA (B-LPA) was generated in vitro by mammalian membranes. These results indicated that a PLA_2_ activity is present in the membrane preparations, suggesting that the sequential actions of PLD and PLA_2_ could generate LPA in this system. These observations provided the basis for the studies reported here. We first addressed the source of the B-LPA generated when B-PC was used as substrate.

The PC12K cell line was used as the principal model system in the current study. As will be presented later, all of the cell lines used were similarly able to generate LPA in membrane preparations. Previous work in our lab established that PC12K cells express only PLD2, while Rat-1 HIR, A7r5, and PC-3 cells express both PLD1 and PLD2 [48]. PLD2 is the only PLD activity detected in membrane preparations with the in vitro assay used herein. The fluorescent metabolites generated in this assay were identified based on their relative mobilities on TLC [53,58]. 

**Figure 1:**
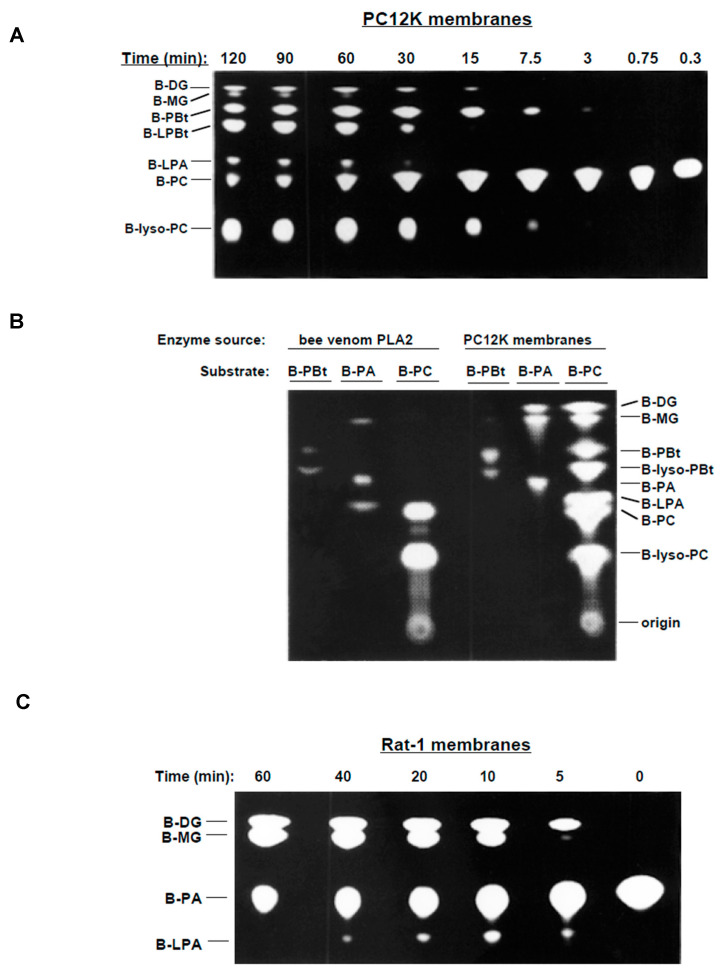
Metabolism of B-PC in PC12K membranes. (**A**) PC12K cell membranes were incubated in vitro for the indicated times with the fluorescent substrate, B-PC, in the presence of 0.5% butanol. Reaction products were separated by TLC and imaged using a FluorImager (reverse contrast). (**B**) PC12K membranes and PLA_2_ from bee venom were incubated with the indicated substrates for 60 min in the presence of 0.5 buranol. B-PA and B-PBt were generated by incubating B-PC with plant PLD in the presence of butanol, followed by TLC purification. Reaction products were assessed as described for (**A**). (**C**) Rat-1 cell membranes were incubated for the indicated times with B-PA in the absence of butanol. Reaction products were assessed as described for (**A**). For all panels, results are representative of three independent experiments.

We examined the kinetics of the generation of the various fluorescent phospholipase metabolites in more detail. A time course of the reaction of PC12K membranes with B-PC in the presence of butanol is shown in Figure 1A. The PLD transphosphatidylation product, phosphatidylbutanol (B-PBt), was detected as early as 3 min. B-Lyso-PC, the PLA_2_ product, appeared by 7.5 min. A product migrating at the position of BODIPY-LPA was detected after 30 min.

The reactions potentially involved in producing each fluorescent B-PC metabolite are as follows. B-PBt is directly generated from B-PC by the PLD transphosphatidylation reaction in the presence of butanol. B-Lyso-PBt (LPBt) can be generated from B-PBt by a PLA_2_ present in membranes [53,58]. B-Lyso-PC is produced when B-PC is hydrolyzed by PLA_2_. The small amounts of BODIPY-diglyceride (B-DG) produced likely arise from the action of an LPP on B-PA. BODIPY-monoglyceride (B-MG), which appeared late in the reaction, could be generated via several routes, including: (1) from B-LPA, via LPP; (2) from B-PA, via LPP/DG lipase; or (3) from B-lyso-PC, via lyso-PLD/LPP. LPA, the product of major interest here, could be generated from PA by a PLA_2_ or from lyso-PC by a lyso-PLD. The pathways generating LPA were further investigated.

We considered whether LPA was being produced from PA. B-PA was not detected as a reaction product at any time (Figure 1A), consistent with previous results using this assay system [53,58]. There are two possible explanations for this result. First, transphosphatidylation (yielding phosphatidylalcohol, rather than PA) is the predominant reaction for mammalian PLD2 in this assay system. In contrast, yeast and plant PLDs generate phosphatidylalcohols and PA in approximately equal amounts under the same conditions [59]. The second possibility is that B-PA is rapidly metabolized, resulting in little or no accumulation. B-PA could be hydrolyzed to B-DG by an LPP. Alternatively, B-PA could be converted to B-LPA by PLA_2_ or LPAAT. These possibilities will be addressed below.

Additional reactions were considered as a potential source of B-LPA. Removal of the BODIPY-fatty acid at the 1-position of B-PC via PLA_1_ cannot generate BODIPY-labelled products via transacylation because B-PC has a 1-alkyl linkage. Phosphorylation of B-MG by MG kinase to generate B-LPA was excluded as a possibility because phosphate donors (e.g., ATP) were not added to the reaction. The possibility of acyl transfer of the substituent at the *sn*-2 position, a reaction that can be dependent or independent of CoA [60], was considered. One CoA-dependent acyltransferase, LPAAT, can generate LPA directly from PA in a reverse reaction [43]. iPLA_2_ has also been reported to exhibit transacylase activity [61]. However, we were not able to show that CoA-independent acyl transfer of a BODIPY-fatty acid occurs under the conditions employed The potential role of LPAAT, an acyl-CoA-dependent enzyme, will be addressed below.

### 3.2. PA-PLA_2_ Activity

Several strategies were employed to determine whether the PLA*_2_* activity present in membrane preparations was involved in LPA generation. First, B-PA and B-PBt were generated from B-PC using plant PLD and then utilized as substrates in vitro (Figure 1B). When B-PBt was incubated with bee venom PLA_2_, B-lyso-PBt was generated. Similarly, in PC12K membranes, B-PBt was converted to B-lyso-PBt and B-MG. These results confirmed that the BODIPY-phosphatidylalcohol served as a substrate for PLA_2_, consistent with our previous findings [53,58].

Metabolism of B-PA was examined (Figure 1B). Bee venom PLA_2_, used as a positive control, hydrolyzed B-PA to generate B-LPA and B-MG. The production of MG suggested that the bee venom PLA_2_ preparation contained phosphatases capable of dephosphorylating B-LPA. Further tests using alternative substrates and other PLA2 preparations were consistent with this conclusion).

With respect to mammalian cells, PC12K membranes converted B-PA to B-DG (Figure 1B, consistent with the presence of LPP activity. B-MG was also observed; this product may have been derived from either B-LPA (via an LPP) or B-DG (via a DG lipase). However, production of B-LPA was not observed in the 60 min incubation with B-PA. To test whether B-LPA could be detected at earlier times, Rat-1 HIR membranes were incubated with B-PA in a time course experiment (Figure 1C). Small amounts of B-LPA appeared as early as 5 min but disappeared by 60 min. The decline in B-LPA is likely due to a combination of metabolism of B-LPA (e.g., to B-MG) and instability of PA-PLA_2_ (i.e., not all phospholipases maintain their activity during a prolonged incubation). The relative kinetics and levels suggest that most of the B-MG detected in Figure 1C originated from deacylation of DG. Thus, the major route for metabolism of B-PA is to B-DG (via LPPs) and not to B-LPA (via PLA_2_). In striking contrast to the minor amounts of B-LPA generated from B-PA (Figure 1C, B-LPA was a major product when B-PC was substrate (Figure 1A–C). Taken together, these results suggested that an additional enzymatic pathway other than PLD/PLA_2_ was largely responsible for generating B-LPA in membrane preparations.

All of the experiments discussed above were conducted in the absence of divalent cations and in the presence of chelators. Consistent with this conclusion, we were unable to detect calcium-stimulated PLA_2_ activity in PC-3 cytosol or membranes in our assay system. This result is consistent with the fact that cPLA_2_ is selective for phospholipids containing arachidonic acid at the *sn*-2 position. PAF acetylhydrolase, a calcium-independent PLA_2_, prefers short acyl substituents (e.g., <4 carbons) [62]. However, since B-PC has a 9-carbon substituent at the *sn*-2 position, it is not expected to be a good substrate for either cPLA_2_ or PPAF-AH. Thus, we conclude that the membrane PLA_2_ analyzed herein is a calcium-independent form (e.g., iPLA_2_).

### 3.3. Lyso-PLD Activity

**Figure 2:**
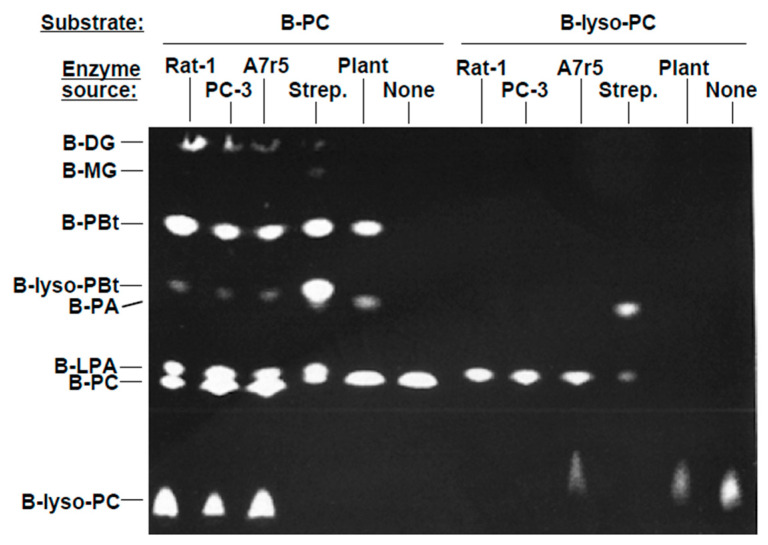
Lyso-PLD activity in cell membranes. Plant PLD, *Streptomyces* PLD, or membranes from Rat-1 HIR, PC-3, or A7r5 cells were incubated with B-lyso-PC or B-PC for 60 min. B-lyso-PC was generated via reaction of B-PC with bee venom PLA_2_. All results were obtained within the same experiment. Reactions using B-PC as substrate were performed in the presence of 1% (*v*/*v*) butanol. The reaction products were separated by TLC and imaged using a FluorImager (reverse contrast). Results are representative of three independent experiments.

The results presented above suggested an alternative route for LPA production in membranes. B-Lyso-PC, a prominent product in the in vitro reactions, could potentially be converted to B-LPA via action of a lyso-PLD. We therefore tested for lyso-PLD activity in the membrane preparations. As substrate, we initially used B-lyso-PC enzymatically generated from B-PC. As shown in Figure 2, membranes prepared from Rat-1 HIR, PC-3, and A7r5 cells generated B-LPA from B-lyso-PC. Complete degradation of B-lyso-PC was seen in many cases, indicating that the amount of substrate was limiting. Cabbage PLD did not generate B-LPA from B-lyso-PC. It has been reported that PLD from *Streptomyces chromofucus* has lyso-PLD activity [63]; this was confirmed by the ability of this enzyme to produce B-LPA from B-lyso-PC. In addition, a novel product that migrated close to B-PA was generated in the reaction of *S. chromofucus* PLD with B-PC. This product may represent cyclic-LPA, which can be produced by *S. chromofucus* PLD [63]. Overall, the results indicated that lyso-PLD activity was present in mammalian membranes.

**Figure 3:**
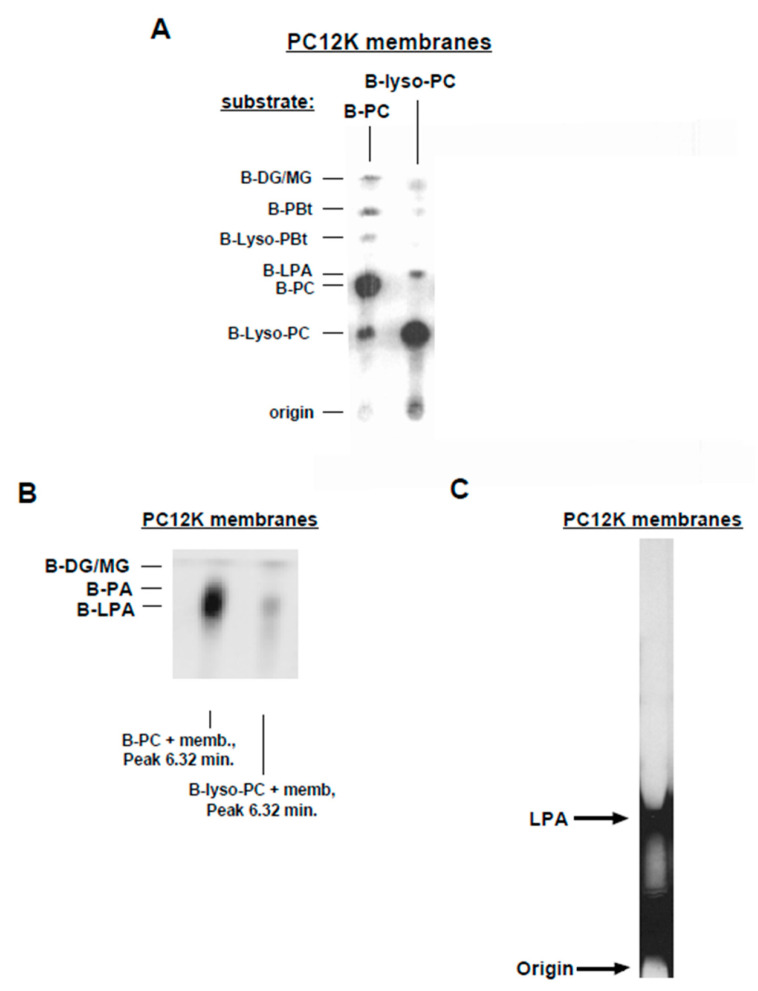
Verification of LPA production. (**A**) PC12K cell membranes were incubated in vitro with either B-PC or commercial B-lyso-PC. Products were separated through TLC and imaged using FluorImager (regular contrast). (**B**) Reaction products were generated as described in (**A**) and then separated using HPLC. B-LPA, generated by purified phospholipases, eluted at 6.32 min (see text). The peaks generated in the two reactions were collected, concentrated, and separated by TLC. (**C**) PC12K membranes were incubated in vitro with [^3^H]-lyso-PAF as substrate. Products were separated on oxalate-impregnated TLC plates and then imaged using autoradiography. The position of LPA is indicated. Results are representative of three independent experiments.

**Figure 4:**
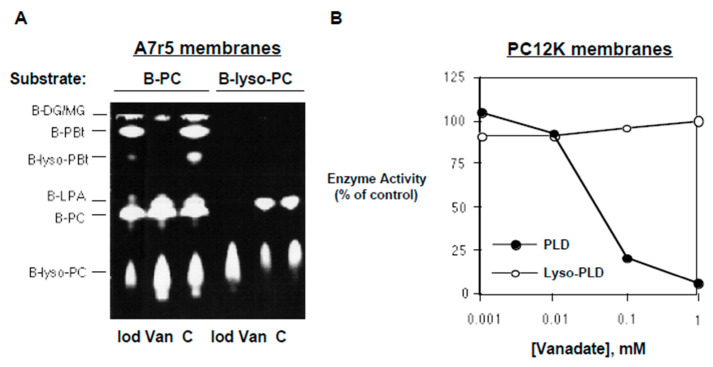
Effects of vanadate and iodate on phospholipase activities. (**A**) A7r5 membranes were incubated with either B-PC or B-lyso-PC in the absence (“C”) and presence of 1 mM of sodium vanadate (“Van”) or sodium iodate (“Iod”). B-lyso-PC was generated enzymatically (i.e., B-PC incubated with bee venom PLA_2_). Products were separated using TLC and imaged using FluorImager (reverse contrast). (**B**) PC12K membranes were incubated with either B-PC (for PLD assay) or commercial B-lyso-PC (for lyso-PLD assay) and the indicated concentrations of sodium orthovanadate. B-PBt and B-LPA were quantified, respectively. Activity is expressed as a percent of that measured without vanadate. Results are representative of three independent experiments.

Reactions utilizing equimolar amounts of B-PC and commercial B-lyso-PC as substrates are presented in Figure 3A. The results again show that B-LPA is generated from B-lyso-PC. To further verify the identity of the product of interest as B-LPA, HPLC was performed as an alternate means of separation. B-PA, produced by incubating plant PLD with B-PC, was incubated with bee venom PLA_2_ to generate B-LPA. The fluorescent products were isolated by TLC and then resolved by HPLC. The retention time of B-LPA in this HPLC system was 6.32 min. B-LPA was effectively separated from B-PC, B-lyso-PC, B-PA, and other reaction products by HPLC, as established utilizing purified phospholipases. We then incubated PC12K membranes with B-PC and B-lyso-PC. The products were separated by HPLC and then analyzed by TLC. The peaks eluting at 6.32 min, generated from either B-PC or B-lyso-PC, migrated identically (Figure 4B). 

LPA acyltransferase (LPAAT) transfers an acyl-CoA to PA, generating LPA. The reverse reaction can also occur, generating PA from LPA [43]. We added a mammalian LPAAT expressed in *E. coli* [56,57] to the in vitro phospholipase assay. Control assays using 18:1 LPA and oleoyl-CoA as substrates established that this preparation was active. However, when the LPAAT preparation was added to the in vitro assay in the presence of PC12K membranes and B-PC, but in the absence of an exogenous acyl-CoA, B-LPA was not generated. As discussed above, acyl acyltransferases do not appear to utilize BODIPY-labeled substrates. 

As an additional approach to establish that lyso-PLD activity was present in mammalian membranes, [^3^H]-lyso-PAF was used as a substrate for the in vitro reaction. Like B-PC, [^3^H]-lyso-PAF is an alkyl-linked choline phospholipid. Products were separated using TLC, as described previously [53], and visualized using autoradiography. PC12K cell membranes generated [^3^H]-LPA from [^3^H]-lyso-PAF (Figure 3C), confirming that lyso-PLD was active under our reaction conditions.

Several additional observations suggested that the membrane lyso-PLD was distinct from PLD, as expected. First, a transphosphatidylation product was not generated by mammalian membranes when B-lyso-PC was used as substrate in the presence of 0.5–2% butanol (e.g., Figure 3A). Thus, unlike PLD, lyso-PLD does not catalyze a transphosphatidylation reaction. Second, we qualitatively observed that lyso-PLD activity was more sensitive to freeze-thawing than was PLD activity. Lyso-PLD activity was substantially decreased in membranes that had been frozen for one week, while PLD activity was maintained. Third, PLD activity was completely inhibited in the presence of 8% dimethylsulfoxide (DMSO), while lyso-PLD was relatively insensitive. The differences between PLD and lyso-PLD activities were further explored.

### 3.4. Inhibitors of PLD and PLA_2_

During the course of our studies, we sought to identify agents that would interfere with PLD or lyso-PLD activity. We found that two inorganic compounds could act as phospholipase inhibitors in vitro. First, we observed that sodium orthovanadate (1 mM) caused profound inhibition of PLD activity, as indicated by decreased production of B-PBt (Figure 4A). In contrast, vanadate did not inhibit lyso-PLD or PLA_2_ activity in the in vitro assay. The IC_50_ for vanadate was approximately 30 µM in mammalian membranes (Figure 4B). The specificity of the effect of vanadate was examined. In screening assays, ompounds that did not inhibit PLD when used at concentrations of 1–10 mM included potassium iodate (Figure 4A) as well as sodium tungstate, sodium oxalate, potassium oxalate, sodium molybdate, magnesium sulfate, copper sulfate, zinc sulfate, magnesium chloride, β-glycerophosphate, and para-nitrophenol phosphate. The effects of iodate on PLD and lyso-PLD activities, also visible in Figure 4A, will be discussed below.

**Figure 5:**
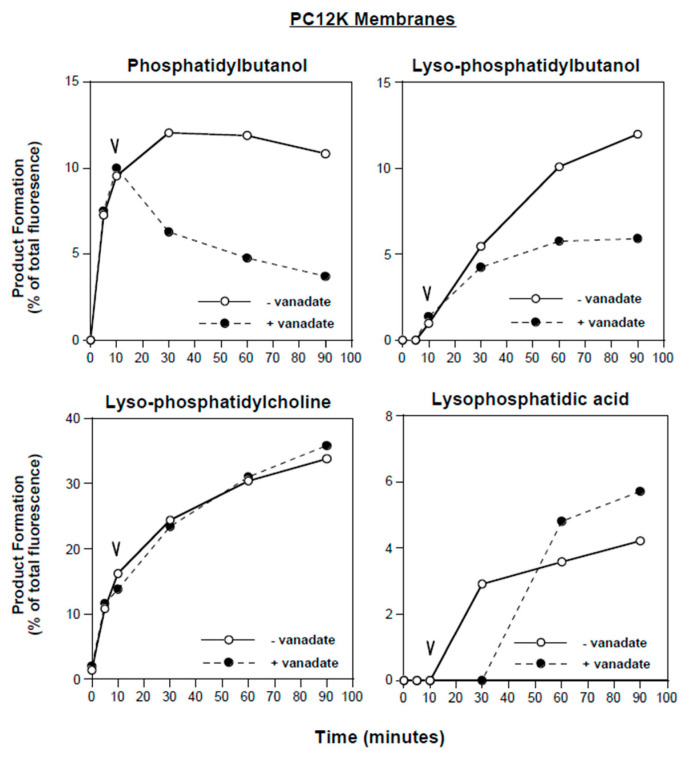
Effects of vanadate on phospholipase reaction kinetics. PC12K membranes were incubated with B-PC for 10 min, at which time either 1 mM sodium orthovanadate or vehicle (H_2_O) was added. The progress of the reaction was followed using TLC, with quantification of the indicated products. Each species is expressed as a percent of total fluorescent products. Results shown are from single experiments representative of three independent experiments.

The ability of vanadate to selectively inhibit PLD was further confirmed in a kinetic study using PC12K membranes. The in vitro reaction was allowed to proceed for 10 min prior to addition of 1 mM sodium vanadate (Figure 5). In the absence of vanadate, B-PBt accumulation reached a plateau after 10 min, while B-lyso-PBt (PLA_2_ product) continued to accumulate. Following addition of vanadate, B-PBt levels declined; B-lyso-PBt production continued but at a reduced rate. In contrast, vanadate did not affect lyso-PC accumulation, consistent with a lack of inhibition of PLA_2_. Production of LPA was not significantly affected by vanadate, confirming that LPA is not generated via PLD activity in this assay system.

The second inorganic inhibitor identified was potassium iodate. Iodate inhibited the production of B-LPA from B-lyso-PC (Figure 4A), indicating inhibition of lyso-PLD activity. Consistent with this conclusion, in reactions utilizing B-PC as substrate (Figure 4A), iodate suppressed the generation of B-lyso-PC and B-lyso-PBt. In contrast, iodate had no effect on PLD activity.

**Figure 6:**
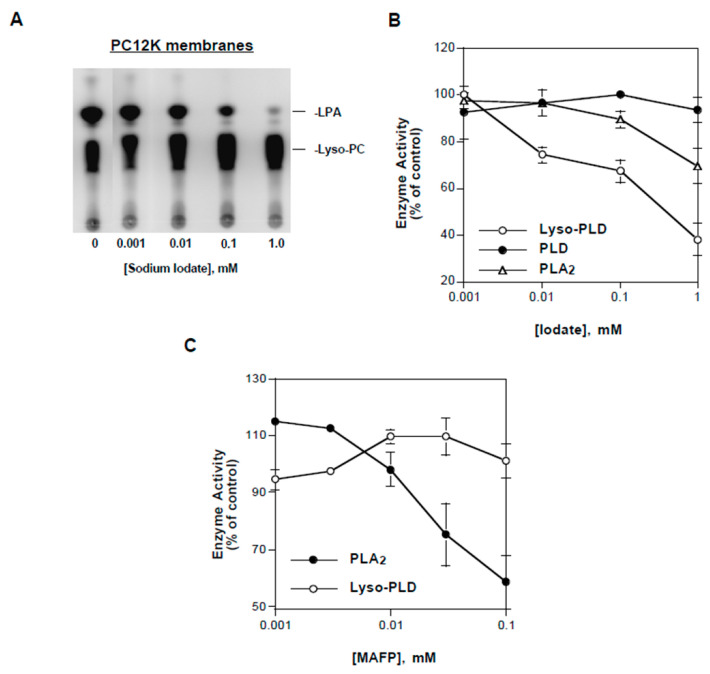
Effects of iodate on phospholipase activities. In (**A**), PC12K membranes were incubated with commercial B-lyso-PC in the presence of the indicated concentrations of sodium iodate. Products were separated using TLC and imaged using FluorImager (regular contrast). Results are representative of three independent experiments. In (**B**), PC12K membranes were incubated with B-PC and 1% (*v*/*v*) butanol (for PLD and PLA_2_ assays) or commercial B-lyso-PC (for lyso-PLD assay) in the presence of the indicated concentrations of sodium iodate. Following TLC separation, levels of B-PBt (for PLD), B-lyso-PBt (for PLA_2_), and B-LPA (for lyso-PLD) were quantified. Activity is expressed as a percent of that measured without iodate. Data points represent mean ± S.D. of values from two separate experiments. In (**C**), PC12K membranes were incubated for 1 h with either B-PC (for PLA_2_ assay) or B-lyso-PC (for lyso-PLD assay) in the presence of the indicated concentrations of MAFP. Following TLC separation, B-lyso-BPC (for PLA_2_) and B-LPA (for lyso-PLD) were quantified. Activity is expressed as a percent of that measured without MAFP. Data points represent mean ± S.D. of values from duplicate reactions in the same experiment.

A concentration–response study of the effect of iodate on lyso-PLD activity is shown in Figure 6A. Quantified data from a similar experiment are presented in Figure 6B. The IC_50_ for iodate on lyso-PLD was approximately 300 µM, while its IC_50_ for PLA_2_ was greater than 1 mM. Iodate had no significant effect on PLD activity at concentrations up to 1 mM.

In view of the fact that iodate inhibited both PLA*_2_* and lyso-PLD, we tested whether a verified PLA_2_ inhibitor had similar effects. Methyl arachidonyl fluorophosphonate (MAFP), an inhibitor of calcium-dependent and calcium-independent PLA_2_s [64], suppressed PLA_2_ activity in PC12K membranes, as evidenced by a decrease in B-lyso-PC when B-PC was the substrate (Figure 6C). In contrast, lyso-PLD (with B-lyso-PC as substrate) was not inhibited by MAFP. These data suggested that PLA_2_ and lyso-PLD activities are conferred by separate proteins.

### 3.5. Further Characterization of Lyso-PLD Activity

**Figure 7:**
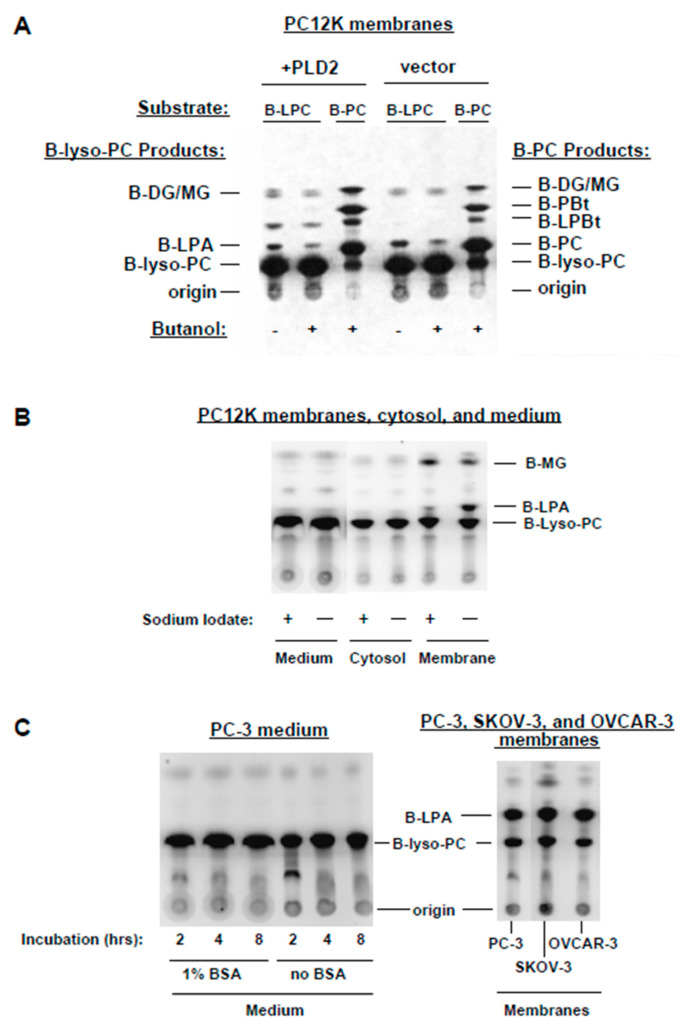
Localization of lyso-PLD activity. In (**A**), PC12K cells were transiently transfected with either empty vector or vector containing a human HA-PLD2 construct. Membranes from the cells were incubated with either B-PC (for PLD assay) or commercial B-lyso-PC (for lyso-PLD assay). Reaction products were separated through TLC and imaged using FluorImager (regular contrast). In (**B**), lyso-PLD activity was assayed, using B-lyso-PC as substrate, in membranes, cytosol, and cell culture medium from the same dish of PC12K cells. Cell and medium samples were equalized for total protein. The assay was performed in the absence and presence of 1 mM of iodate. Products were imaged using FluorImager (regular contrast) following TLC separation (a separate plate was run, in parallel, for culture medium). In (**C**), serum-starved PC-3 cells were incubated for the indicated times in the absence or presence of 1% BSA. Lyso-PLD activity was assessed in samples of the serum-free medium using B-lyso-PC as substrate. In the same experiment, lyso-PLD activity was assayed in membranes from PC-3, SKOV-3, and OVCAR-3 cells (equalized for protein). Products were imaged using FluorImager (regular contrast) following TLC separation.

As an additional strategy to test whether PLD played a role in LPA production, in the membrane preparations we over-expressed PLD2 in PC12K cells. Transient transfection resulted in elevated PLD2 activity in membranes, as seen with B-PC as substrate in the presence of butanol (Figure 7A). However, neither LPA production from B-PC nor lyso-PLD activity (using B-lyso-PLD) was increased in membranes from transfected cells. Two additional features illustrated in Figure 7A are as follows. First, a novel unidentified reaction product (migrating below lyso-PBt) was increased in membranes from PLD2-transfected cells using B-lyso-PC as substrate. Second, lyso-PLD does not perform a transphosphatidylation reaction (compare results using B-lyso-PC as substrate, with butanol vs. without butanol). Alcohols consistently reduced the formation of B-LPA in the membrane assay, as illustrated here, but an alternate reaction product was not observed. Thus, the data presented in Figure 7A confirm that PLD2 and lyso-PLD are distinct activities and that PA does not contribute to LPA production in this assay system.

The localization of the lyso-PLD activity was examined. In PC12K cells (Figure 7B), lyso-PLD activity was found only in membranes, where it was inhibited by iodate. Such activity was not detected in cytosol or in cell culture medium. PC-3 cells were incubated up to 8 h in serum-free medium, in the absence and presence of 1% bovine serum albumin, to determine whether extracellular lyso-PLD might accumulate in the medium over time (Figure 7C). PC-3 cells were used for this experiment because these cells can generate extracellular LPA [8]. However, lyso-PLD activity was not detected in the medium. In the same experiment, as a positive control, membrane lyso-PLD activity in PC-3 cells (prostate cancer) was directly compared to that in OVCAR-3 and SKOV-3 (ovarian cancer). Similar levels of activity were seen in all three human cell lines.

Lyso-PLD activity present in plasma is conferred by autotaxin (ATX; ecto-nucleotide pyrophosphatase/phosphodiesterase PD-1α) [36,37]. In additional qualitative experiments, we found no evidence that varying concentrations of CaCl_2_, MgCl_2_, CoCl_2,_ or ATP did not stimulate lyso-PLD activity in our assay system, further indicating that the membrane lyso-PLD activity is not autotaxin.

### 3.6. Autotaxin Knockdown Experiments

**Figure 8:**
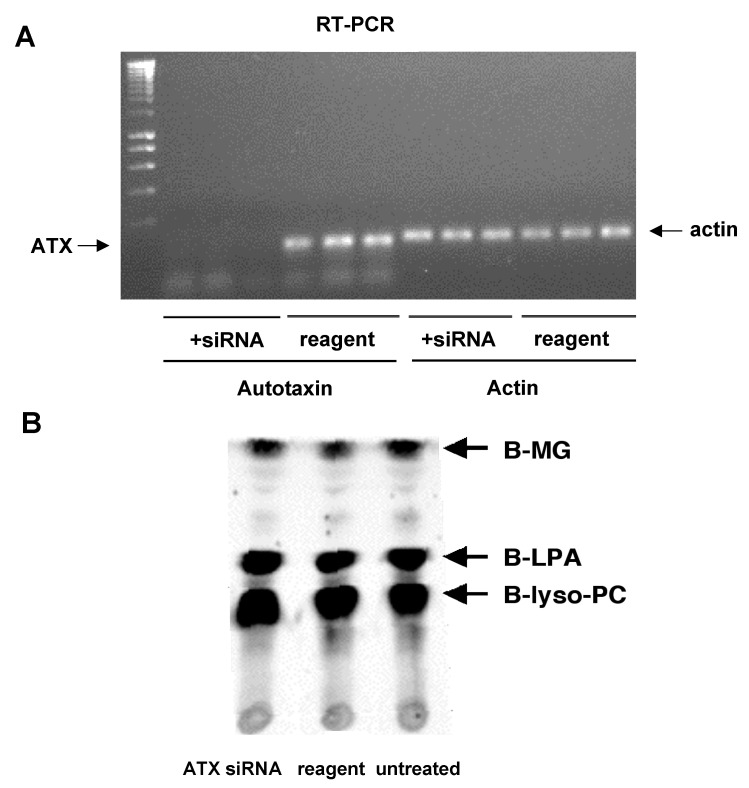
Effects of autotaxin siRNA on membrane lyso-PLD activity. (**A**) OVCAR-3 cells were incubated with siRNA for autotaxin, or with transfection reagent alone, for 72 h. Triplicate dishes were used for each experimental condition. Autotaxin and β-actin mRNAs were amplified through RT-PCR; products were separated on an agarose gel containing ethidium bromide and visualized using UV light. (**B**) An assay for membrane lyso-PLD activity was carried out using membranes from OVCAR-3 cells treated as in (**A**) (autotaxin siRNA; transfection reagent alone; untreated). Reaction products were imaged using FluorImager (regular contrast) following TLC.

To further test whether autotaxin (ATX) was a source of membrane lyso-PLD activity, we conducted a series of experiments utilizing silencing RNA to reduce levels of ATX expression. We first screened for autotaxin mRNA expression in several of the cell lines used in our laboratory, using real-time PCR. We focused our subsequent studies on OVCAR-3 cells which expressed relatively high levels of ATX mRNA A siRNA construct was designed for ATX. Following the addition of this reagent to OVCAR-3 cells, ATX mRNA levels were reduced by eight-fold, as determined using real-time PCR. The success of the RNA interference was confirmed through direct visualization of the RT-PCR products (Figure 8A). The effects of the ATX knockdown on membrane lyso-PLD activity were examined. As shown in Figure 8B, membrane lyso-PLD activity was not decreased after ATX knockdown. These data provide further evidence that the membrane lyso-PLD is distinct from autotaxin.

### 3.7. Substrate Utilization by Lyso-PLD

**Figure 9:**
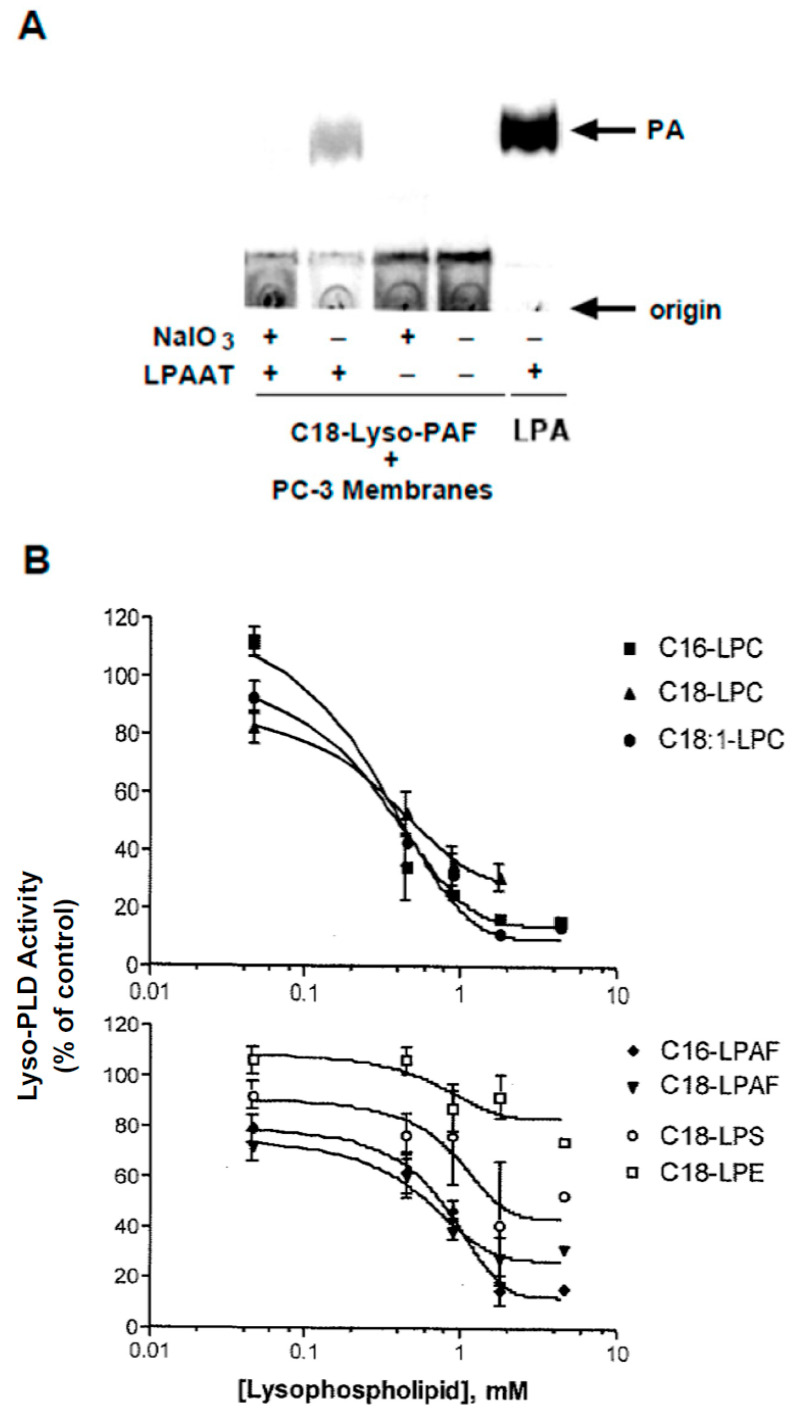
Utilization of naturally occurring substrates by lyso-PLD. (**A**) PC-3 cell membranes were incubated with unlabeled C_18_-alkyl-lyso-PC in the absence and presence of 1 mM sodium iodate. Lipids extracts from the reaction were incubated with LPAAT and [^14^C]-oleoyl-CoA. Product ([^14^C]-PA) was extracted and separated using TLC. The autoradiogram of the TLC plate is shown. The right lane is a positive control in which the first step of the incubation was carried out using buffer alone (no membranes); the lipid extract was incubated in the LPAAT reaction in the presence of exogenous 18:1 LPA (20 pmole). The results are from a single experiment that is representative of three independent experiments. (**B**) In vitro assays for lyso-PLD were carried out using PC-3 membranes with B-lyso-PC as substrate in the presence of varying concentrations of the indicated lyso-phospholipids. The concentration of B-PC in this assay is 0.046 mM; competing lipids were added at ratios of 1:1, 10:1, 20:1, 40:1, and 100:1 with respect to B-PC. Production of B-LPA was assessed as a percent of total fluorescence. The value obtained in the absence of competing phospholipid was set at 100% activity. Curves were fit for single-site competition using GraphPad Prism. Each point represents the mean ± S.D. of values from two independent experiments, except for the highest concentration (4.6 mM), which was tested in a single experiment.

Most of the experiments described above utilized BODIPY-labeled lyso-PC as substrate. To further confirm the ability of lyso-PLD to utilize naturally occurring substrates, LPA production was assessed using a radioenzymatic assay [56]. PC-3 cell membranes were incubated with unlabeled C_18_-lyso-PAF under the same conditions employed for the fluorescent assay. The lipid products were extracted and then incubated with LPAAT in the presence of [^14^C]-oleoyl-CoA. As shown in Figure 9A, [^14^C]-PA was produced, confirming that lyso-PAF had been converted to LPA. When the membrane reaction was carried out in the presence of 1 mM sodium iodate, LPA production was nearly completely inhibited. These findings confirm that lyso-PLD in mammalian membranes is capable of utilizing naturally occurring substrates. 

To further explore substrate specificity, the ability of various lyso-phospholipids to compete with B-lyso-PC for utilization by lyso-PLD was tested using PC-3 membranes. As quantified in the upper panel of Figure 9B, C16-, C18-, and C18:1-lyso-PCs (acyl-linked PCs) were similarly effective in inhibiting B-lyso-PC conversion to B-LPA, with EC_50_ values of ~0.3 mM. In the lower panel, C16- and C18-lyso-PAFs (alkyl-linked PCs) also inhibited B-lyso-PC utilization, with EC_50_ values in the range of 0.4 mM. The results consistently indicated that alkyl-linked PCs were less effective competitors than acyl-linked PCs. The relatively high EC_50_ values probably reflect the fact that the substrate (B-lyso-PC) is not present at saturating concentrations in this assay. Lyso-phosphatidylserine (LPS) was a poor competitor, and lyso-phosphatidylethanol (LPE) was the least effective. Only B-LPA production was affected by the lysophospholipids. These data suggest that membrane lyso-PLD can utilize a variety of naturally occurring lyso-PC species as substrates.

### 3.8. Analysis of GDE4 and GDE7 Expression

**Figure 10:**
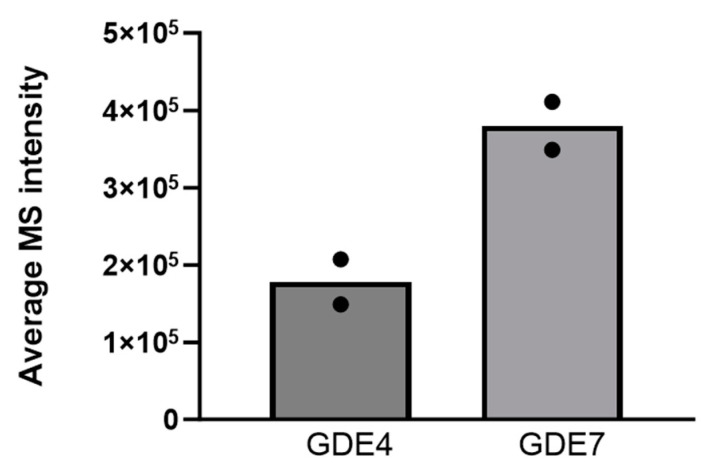
Proteomics analysis of the expression of lyso-PLDs in PC-3 cells. Expression levels of GDE4 and GDE7 were quantified from a global proteomics analysis of serum-starved cells [52]. Each bar represents the mean of values (shown as dots) from duplicate samples in which the proteins were detected.

To determine which of the known membrane lyso-PLDs is expressed in PC-3 cells, one of the cell lines used in this study, we extracted data from a global proteomics analysis of PC-3 cells [52]. For this analysis, cell lysates were prepared from serum-starved PC-3 cells and then subjected to proteolysis followed by LC/MS separation and identification of the protein fragments. Protein abundance was quantified as part of the proteomics analysis. Note that this analysis was performed using whole-cell lysates from serum-starved cells [52], whereas the phospholipase assays were performed using membranes from cells growing in serum. The results show that PC-3 cells express both GDE4 and GDE7 (Figure 10). Each protein was detected in two out of the three replicate samples, satisfying the criterion for verified expression with the rigorous algorithm used for the analysis. Both proteins were expressed at relatively low levels; GDE7 was more abundant than GDE4. 

## 4. Discussion

**Figure 11:**
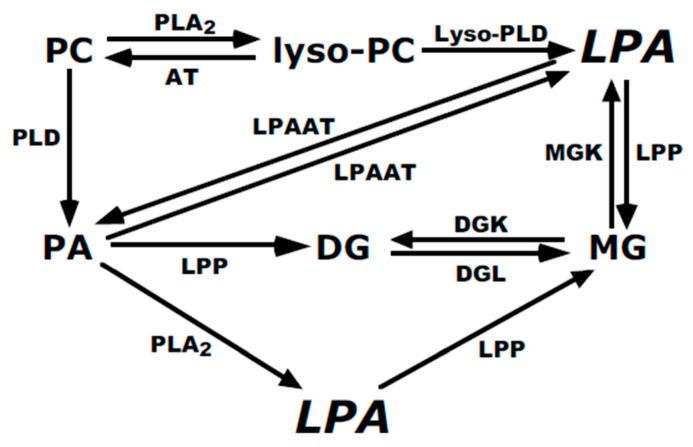
Pathways for LPA generation and metabolism. This scheme depicts enzymatic pathways that potentially participate in LPA generation and degradation. Enzymes that were a primary focus of this work are outlined in bold. The abbreviations used are: AT, acyltransferase; PC, phosphatidylcholine; PA, phosphatidic acid; LPA, lysophosphatidic acid; DG, diglyceride; DGK, diglyceride kinase; DGL, diglyceride lipase; LPAAT, lysophosphatidic acid acyltransferase; LPP, lipid phosphate phosphatase; lyso-PLD, lyso-phospholipase D; MG, monoglyceride; MGK, monoglyceride kinase; PLA_2_, phospholipase A_2_; PLD, phospholipase D.

The phospholipid metabolism pathways considered in this study are depicted in Figure 11. The major conclusion of this work is that a membrane-associated lyso-PLD activity is expressed by a variety of mammalian cell lines.

The studies described herein emphasize the potential role of lyso-PLD activity in LPA generation. Lyso-PLD is the major pathway contributing to LPA production in the membrane preparations used in our study. This enzyme activity remains to be fully characterized. In early reports, one lyso-PLD was reported to require magnesium, to be inhibited by DTT, and to be relatively insensitive to freeze-thawing [20]. Another previously characterized lyso-PLD required calcium and was insensitive to DTT [21]. Neither description applies to the enzyme activity described herein. However, several studies agree that lyso-PLDs can utilize alkyl-linked glycerophosphocholines [21,22,38], consistent with our findings. Other investigators have characterized lyso-PLDs that are secreted from cells. One group identified a lyso-PLD that is secreted by adipocytes and is stimulated by divalent cations [36]. The lyso-PLD activity present in plasma [30,31,34], autotaxin, can utilize both acyl- and alkyl-linked substrates [37,38].

The phosphatase activity of autotaxin is stimulated by divalent cations (e.g., Mg or Co), it but can be measured in their absence [65]. GDE4 and GDE7, membrane-localized lyso-PLDs, are stimulated by Mg [41]. Although the enzyme described herein did not appear to be cation-stimulated, further studies will be needed to fully address this point because our experiments were completed prior to the characterization of GDE4 and GDE7. An advantage of the fluorescent assay used herein, originally developed as a PLD assay, is the ability to visualize changes in the levels of the substrate as well as changes in the levels of all fluorescent metabolites. This led to our initial observation of a lyso-PLD activity in the membrane preparations and also allowed us to characterize the PLA_2_ activity that generates the lyso-PLD substrate.

The membrane lyso-PLD identified in our study is not present in detectable amounts in culture medium. Autotaxin and related type I phosphodiesterases are transmembrane glycoproteins that can act as both ecto- and exo-enzymes [66]. Although catalytically active forms of these enzymes are released into the extracellular space by proteolysis, the intact proteins are present on the plasma membrane as well as on intracellular membranes. However, the results of our siRNA knockdown experiments, along with other lines of evidence, establish that the membrane lyso-PLD described in this study is not autotaxin.

It is likely that the lyso-PLD activity characterized in the current study can be attributed to GDE4 and/or GDE7. These membrane-localized enzymes were characterized as lyso-PLDs subsequent to the completion of the experiments reported herein. We detected lyso-PLD activity in a variety of cell lines, including PC-3 (human prostate cancer) cells. Data derived from a global proteomics analysis of PC-3 cells show that this cell line expresses both GDE4 and GDE7. Our results support the hypothesis that membrane lyso-PLD activity is conferred by the recently identified lyso-PLDs, GDE4, and/or GDE7.

A previous report showed that both GDE4 and GDE7 are widely expressed in mouse tissues; levels were relatively low in ovary, while prostate was not tested [38]. A fluorescent substrate for ATX was recently adapted for use in an assay that detected lyso-PLD activity in membranes from HEK293 (human embryonic kidney) cells transfected with GDE4 and GDE7 and from untransfected LNCaP (human prostate cancer) and MCF-7 (human breast cancer) cells [67]. A more comprehensive analysis of the cellular expression of GDE4 and GDE7 in various cell types, and in normal and pathological situations, is warranted to provide additional perspective on the cellular roles of these enzymes.

The fluorescent assays described herein are particularly useful in that the complete spectrum of reaction products can be readily visualized. In this way, the presence of enzymes that compete for substrate or degrade products can be assessed. BODIPY-lyso-PC is a convenient substrate for assay of lyso-PLD activity. Unique structural features of this particular substrate, which include an alkyl linkage and fluorophore at the *sn*-1 position, are likely to influence the spectrum of reaction products observed. The reaction conditions (e.g., the absence of divalent cations and the presence of detergent) are also expected to influence the range of metabolites. Thus, as is the case for any in vitro assay, the relative predominance of activities does not necessarily reflect the situation existing in vivo. With respect to potential physiologic substrates, our data show that membrane lyso-PLD can utilize naturally occurring acyl- and alkyl-PCs.

Production of phosphatidylalcohols is widely used to measure PLD activity in intact cells. The advantages of these metabolites are that they are only generated after alcohol addition and that they are less susceptible to metabolism than PA. Our results confirm that phosphatidylalcohols serve as substrates for PLA_2_. Metabolism of phosphatidylethanol (PEt) by intact cells was reported [68,69,70], but it appears to be minimal in some cell types [71]. Hydrolysis of PEt by an unspecified PLA*_2_* was reported previously [72].

Multiple isoforms of PLA_2_ have been identified in mammalian cells, with more than one form involved in agonist-induced arachidonate release [65]. The role of secretory PLA*_2_* in LPA generation has been considered with respect to inflammation [73]. PA-utilizing PLA*_2_* activity was originally studied in human platelets, where it participates in LPA generation (reviewed in [74]). A PA-specific PLA_1_ was also characterized [75]. Since LPAs can have a substituent at either the *sn*1- or *sn*2-position, PLA_1_ may play a role in LPA production. The results presented here suggest that many cell types have the potential to produce LPA. It is likely that PLDs, PLAs, LPAAT, and/or lyso-PLD can all participate in LPA generation, with the predominating pathway(s) potentially varying between cell types. The relative contributions of intracellular and extracellular pathways to LPA production, and the access of LPA to its receptors when produced by membrane lyso-PLDs, are of particular interest.

The discovery that vanadate and iodate inhibit mammalian PLD and lyso-PLD activities, respectively, provides useful tools for in vitro experiments. However, studies in our lab using multiple cell types under varying experimental conditions have failed to show that vanadate, pervanadate, or iodate inhibits PLD in intact cells. Pervanadate has been shown by others to activate PLD in intact endothelial cells [76]. Iodate (1 mM) did not decrease LPA levels in intact PC12K cells or medium [Gibbs and Meier, unpublished data], as assessed using a radioisotopic assay for LPA production [54]. The failure of vanadate and iodate to inhibit phospholipases in intact cells may be because sufficient intracellular concentrations of the inhibitors cannot be achieved. Mechanistically, vanadate mimics the transition state of the phosphate group involved in ester hydrolysis, while iodate can bind metal ions involved in catalysis. Vanadate was previously reported to inhibit a PLC from *Bacillus cereus* [77]; iodate can inhibit the same enzyme [78]. Vanadate was previously shown to directly inhibit cabbage PLD [79,80]. To our knowledge, the effects of iodate on GDE4 and GDE7 have not been tested.

Lyso-PLDs represent potential targets for pharmacologic intervention in cancer. Previous work from our group has demonstrated that PC-3 and Du145 prostate cancer cells can generate bioactive LPA species and respond mitogenically to the LPA that they produce [8]. Others have established that multiple cell types, including ovarian cancer cells, can produce LPA [7]. Given the prominent biological activities of LPA, it is a high priority to determine the roles of the different lyso-PLD enzymes in cell signaling. The development of additional inhibitors of LPA production and response, which depends on identification of the relevant enzymes, is an ongoing and important therapeutic goal.

## 5. Conclusions

This study has established that a lyso-PLD activity is present in membrane preparations from a variety of mammalian cell lines. The enzyme(s) utilizes either acyl- or alkyl-linked lysophatidylcholines and can be detected in an in vitro assay using a fluorescent lyso-PC as substrate. The membrane lyso-PLD is distinct from autotaxin, a secreted form of lyso-PLD. Membrane lyso-PLD activity is sensitive to inhibition by iodate, whereas membrane PLD2 activity is inhibited by vanadate. It is likely that membrane lyso-PLD activity is conferred by GDE4 and/or GDE7, membrane lyso-PLDs that are widely expressed. The subcellular localization and functions of these intriguing enzymes are still in the process of being elucidated [81,82,83,84]. The contributions of GDE4 and GDE7 to producing LPA that can activate membrane-localized LPA receptors, GPCRs that mediate a wide range of cellular responses, remain to be elucidated.

## Data Availability

Supporting data are available upon request from the corresponding author.
